# Variance partition that eludes intuition^☆^

**DOI:** 10.1016/j.ssmph.2025.101763

**Published:** 2025-02-17

**Authors:** Jay S. Kaufman

**Affiliations:** Department of Epidemiology, Biostatistics, and Occupational Health, School of Population and Global Health, Faculty of Medicine and Health Sciences, McGill University, Suite 1200, 2001 McGill College Avenue, Montréal, Québec, H3A 1G1, Canada

**Keywords:** Birthweight, Hispanic ethnicity, Multilevel modeling, MAIHDA, Analysis of variance

Human beings are variable in their biological endowments and environmental exposures, but an enduring truth of this variability is that it is continuous rather than discrete (Livingstone, 1962). For epidemiological science, this implies that all human groupings are the products of complex social, political and historical traditions, with no hope of being coherent across the relevant dimensions of biology and environment that may bear on human disease (Mersha & Beck, 2020). Races and ethnicities are widely used in epidemiology to group socially recognized subpopulations with some presumed common traits or experiences, but perhaps no grouping is as perplexing in its arbitrariness as the quaint American construction of the “Hispanic” ethnicity. Enshrined in the Office of Management and Budget (OMB) Directive 15 as the only ethnicity officially recognized in the United States (OMB 1978), “Hispanics” were defined as persons “of Mexican, Puerto Rican, Cuban, Central or South American or other Spanish culture or origin, regardless of race.” This is not an ethnicity in the usually understood sense of a shared culture or identity, and there is no similar ethnic construct for people around the world who speak French or English. Rather it reflects a unique historical and political understanding within the United States in relation to the various other peoples of the Western Hemisphere, and how they would relate to the US as immigrants, laborers and colonial subjects (Nelson & Tienda, 2014).

In this issue of The Journal, Borrell and colleagues examine birthweight differences among about 820,000 singleton births in New York City from 2012 to 2019, considering joint categories of maternal age, race/ethnicity, education, and nativity status (Borrell et al., 2025-a). They have a particular focus on disaggregating the Hispanic/Latina group into 7 subpopulations defined by national origin. Clearly the overall Hispanic/Latina category is impossibly diverse, and so dividing this by ancestral geography is a welcome step toward reducing within-group heterogeneity. The tension in this sort of refinement is always that smaller groups lead to less precise estimates, and thus to a trade-off between more stable means hiding diversity within groups, or less stable means showing diversity between groups, but noisily. Fortunately, these authors employed a relatively new method for balancing these trade-offs, the clever innovation in multilevel modeling called “MAIHDA”, which stands for “Multilevel Analysis of Individual Heterogeneity and Discriminatory Accuracy”. This is an approach proposed by Juan Merlo roughly 10 years ago (Merlo, 2014) in which each intersectional stratum is allowed its own random intercept. First the population is cross-classified by all the pre-determined categories of interest; in this case: maternal age, race/ethnicity, education, and nativity status. For Borrell and colleagues, this amounted to 4 age groups x 11 race/ethnicity categories x 3 education groups x 2 nativity groups = 264 potential strata, or which 257 were populated. These ranged in size from n = 1 to n = 78,023, with about 20% of the cells having fewer than 50 observations. A fixed effects model would therefore be disastrous, with 263 indicator variables and many imprecise means. Fitting only the main effects terms would force additive joint effects without any substantive justification. Merlo's solution of 256 random intercepts is brilliant, because it requires only a small number of parameters and allows sparse cells to be shrunk toward the grand mean, guaranteeing better overall prediction (Bell et al., 2019).

The authors found that the overall birthweight mean was 3266 g, with a standard deviation of 534 g, implying that about 95% of births fall between 2200 and 4300 g. When considering the 7 distinct Hispanic ancestries in addition to the 4 non-Hispanic race groups in a null-random intercept model, 2.7% of the variation in birthweight was found to lie between the 257 intersection strata, whereas 97.3% of the variation in birthweight was within these groups. This shows that the overwhelming majority of the variation is not attributable to age, education, race/ethnicity or nativity, but rather to other unmeasured factors that varied within these strata, such as behaviors (e.g. diet, smoking), environments (e.g. air pollution, noise), genetics and medical care. Moreover, when main effects dummies (3 for age, 10 for race/ethnicity, 2 for education and 1 for nativity) were added to the model, these absorbed over 80% of the between-groups variance, leaving a residual between-groups variance of only 0.5%. This final residual represents the contribution of all interaction terms between factors that would arise from non-additive joint effects. In this application, such non-additive interactions turn out to be trivial. The result is very clear: knowing that a woman is Puerto Rican or 35 years old or a high school graduate gives very little information about the birthweight of her child. The mean for Puerto Rican women is 105 g lower than for non-Hispanic white women, but since the range within Puerto Rican women is so wide, this information about the group mean is practically useless. Why? Because Puerto Rican birthweights are not located at the mean value for their group; they are located in a wide distribution around that mean, from roughly 2100–4200 g. Knowing something about the average of all Puerto Rican woman tells you almost nothing about any one specific Puerto Rican woman. That distinction was the beauty and the power of Merlo's statistical invention, and why the name of the tool contains the terms “Discriminatory Accuracy” (Merlo et al., 2017).

To illustrate how woefully uninformative are these stratifications, lets say that US-born 20–29 year old non-Hispanic white women with less than high school education have a mean birthweight of 3300 g and a standard deviation of 530 g (Table 3), whereas mainland-born Puerto Rican women with the same age and education level have a mean birthweight that is 105 g less, with the same standard deviation. Draw a birth at random from each distribution and then ask: what is the probability that the Puerto Rican woman has the lower birthweight in the pair? If the means were equal, that probability would be 50.0%. With the 105 g difference, the probability is only 55.6%. Almost half the time, it is the non-Hispanic white woman with the lower birthweight. This is why the ethnic information is essentially useless for inferring the value for the outcome of any individual. Despite the apparently large gap between their means, about 42% of Puerto Rican births are above the average for Non-Hispanic white women and likewise, about 42% of births for white woman are below the Puerto Rican average.

The authors expertly analyzed an impressive dataset, applying the MAIHDA model skillfully and informatively, and clearly demonstrating that race and ethnicity are not the important drivers of differential birthweight in this setting, relative to the vastly more important play of unseen forces operating within each of the 257 strata. But it is curious that the discussion section of the paper dwells on the racial and ethnic dimension as though it is the major story. It is as if they don't want to view the cup as 97% empty, but instead prefer to focus on it being 3% full. And keep in mind that this 3% of variance is what is due jointly to age, education, nativity and race/ethnicity; the race/ethnicity contribution is only some fraction of that 3%.

The authors stress that in the model with Hispanic subgroups, the difference between strata with the highest and lowest predicted birthweight means was 370 g, which seems like a large disparity. But this is a disparity that applies to the difference between n = 119 US-born Asian teenage mothers with low education versus n = 3926 US-born 30–39 year old white mothers with low education. So while 370 g seems like a big gap, it is a big gap for just ∼4000 births out of 820,000, or less than 0.5% of the sample. The other 99.5% have smaller gaps, the vast majority not even significantly different from the overall mean, as can be seen in Figure 1B. The grand mean line in Figure 1B is shown at 3230 g, and about 2/3 of the 820,000 births in the dataset are in strata with predicted means that fall within 100 g of this overall average. Nonetheless, the authors insist that this 3% of variance is “meaningful”. Sure, of course it's meaningful, in the same sense that $3 out of $100 is meaningful, but not as meaningful as the other $97.

The authors conclude that their findings “underscore how systems of oppression/privilege such as racism and xenophobia may act as a matrix of dominance to create and perpetuate health inequities.” While this statement is surely true, I struggle to see this interpretation supported anywhere in the quantitative results of this paper. Rather, what this model seem to be trying to tell us is that we have not yet even begun to understand what makes some babies larger and some smaller, given that the vast majority of variation in this model was not attributed to any measured variable. It's very hard to interpret residual variance, because it is literally unexplained. Methodologists have warned for decades that percent variance explained is not a measure of population impact (Greenland et al., 1986). This perspective does allow for effects to be important even when the R^2^ is small, for example if an adverse outcome is rare. Consider infant mortality, which occurs in this population at a frequency of roughly 4–5 per 1000 births. Low birthweight is the most significant risk factor for infant mortality, and an exposure that caused some number of births to be severely underweight could have a large proportionate impact on the frequency of infant deaths, even while having a trivial impact on the overall birthweight distribution. To demonstrate this more definitely, however, the authors might find it more illuminating to study the occurrence of these rare adverse events, like infant deaths, rather than the average birthweight across the entire population, the vast majority of which is healthy in all strata. There is much more to be learned about the disparities presented in this article, and I hope that these authors will continue to use this data resource and their skill with the MAIDHA model to carry this story forward with further insights.

## Funding sources

No funding was received for the production of this manuscript.

## Declaration of interest statement

I received no financial support for this work.

## Data Availability

No data was used for the research described in the article.

## References

[bib1] Bell A., Holman D., Jones K. (2019). Using shrinkage in multilevel models to understand intersectionality: A simulation study and a guide for best practice. Methodology.

[bib2] Borrell L.N., Nieves C., Evans C.R. (2025). Examining variation within Hispanic ethnicity: An intersectional MAIHDA analysis of birthweight inequities in New York City, 2012-2019. SSM-Population Health.

[bib3] Directive No. 15 (1978). Race and ethnic standards for federal statistics and administrative reporting. Federal Register.

[bib4] Greenland S., Schlesselman J.J., Criqui M.H. (1986). The fallacy of employing standardized regression coefficients and correlations as measures of effect. American Journal of Epidemiology.

[bib5] Livingstone F.B. (1962). On the non-existence of human races. Current Anthropology.

[bib6] Merlo J. (2014). Multilevel analysis of individual heterogeneity—a fundamental critique of the current probabilistic risk factor epidemiology. American Journal of Epidemiology.

[bib7] Merlo J., Mulinari S., Wemrell M., Subramanian S.V., Hedblad B. (2017). The tyranny of the averages and the indiscriminate use of risk factors in public health: The case of coronary heart disease. SSM-population health.

[bib8] Mersha T.B., Beck A.F. (2020). The social, economic, political, and genetic value of race and ethnicity in 2020. Human Genomics.

[bib9] Nelson C., Tienda M. (2014). Challenging fronteras.

