# Supplementary material for: Variance partition that eludes intuition
Source: SSM Popul Health. 2025 Feb 17;29:101763. doi: 10.1016/j.ssmph.2025.101763 (PMC11880606; doi:10.1016/j.ssmph.2025.101763)
Supplement: Multimedia component 1 [file mmc1.docx]

Not intended for publication, but rather just for fact checking, this is the proof of the quantitative probability statements included in my commentary:

Let X be the observation from white non-Hispanic women . X ~ N(3300, 530^2^). Let Y be the observation from Puerto Rican women Y ~ N(3195, 530^2^). We are interested in the probability that Y < X, or equivalently, X - Y > 0. Define random variable D = X - Y. Since X and Y are independent normal random variables, their difference D is also normally distributed. The mean of D (μ_D_) = μ_X_ - μ_Y_ = 3300 - 3195 = 105. The variance of D (σ_D_^2^) = σ_X_^2^ + σ_Y_^2^= 530^2^ + 530^2^ = 2 * 530^2^. Therefore the standard deviation of D (σ_D_) = sqrt(2 * 530^2^) = 530 * sqrt(2) ≈ 749.5. So, D ~ N(105, (530*sqrt(2))^2^). We need to find P(X - Y > 0) = P(D > 0). To do this, standardize D: Z = (D - μ_D_) / σ_D_ = (0 - 105) / (530 * sqrt(2)) ≈ -0.14. Then find P(Z > -0.14), which is equal to 1 - P(Z < -0.14). Using a standard normal distribution table:

P(Z < -0.14) ≈ 0.4443. Therefore, P(D > 0) = 1 - 0.4443 ≈ 0.5557. The probability that a random Puerto Rican birthweight is smaller than a random non-Hispanic white birthweight for women of the same age, nativity and education, is approximately 0.556, or 55.6%.

What is the proportion of Puerto Rican birthweights that lies above the mean for white non-Hispanic women? This is the same as the proportion of white non-Hispanic birthweights that lies below the mean of Puerto Rican women. Puerto Rican birth weights are normally distributed with mean μ_Y_ = 3195 and standard deviation σ_Y_ = 530. So find P(Y > 3300), where Y is a random variable representing an observation from the Puerto Rican population. Standardize Y: Z = (Y - μ_Y_) / σ_Y_ = (3300 - 3195) / 530 = 105 / 530 ≈ 0.198

Now, we want P(Z > 0.198). Using a Z-table: P(Z > 0.198) = 1 - P(Z < 0.198) ≈ 1 - 0.5783 ≈ 0.4217. The same number applies to the proportion of white non-Hispanic birthweights that lies below the mean of Puerto Rican women.
